# The Application Value of Syndecan-2 Gene Methylation for Colorectal Cancer Diagnosis: A Clinical Study and Meta-Analyses

**DOI:** 10.3389/fmed.2022.753545

**Published:** 2022-03-15

**Authors:** Congbo Yue, Yaping Zhang, Yanlei Wang, Zhenhong Zhang, Mengjiao Zhang, Huayang Wang, Wendan Chen, Ziqi Shang, Yiwei Xin, Xin Zhang, Yi Zhang

**Affiliations:** ^1^Department of Clinical Laboratory, Qilu Hospital of Shandong University, Jinan, China; ^2^Department of General Surgery, Qilu Hospital of Shandong University, Jinan, China; ^3^Jinan AXZE Medical Test Laboratory, Jinan, China

**Keywords:** droplet digital PCR, diagnostic value, colorectal cancer, SDC2 gene methylation, biomarker

## Abstract

**Objective:**

Syndecan-2 (SDC2) methylation has been previously reported as a sensitive biomarker for the early detection of colorectal cancer (CRC). Droplet digital PCR (ddPCR) is the latest development of PCR technology. It can accurately detect and quantify the target sequence of nucleic acid. ddPCR is widely used in research and clinical diagnosis. In the present study, we aimed to develop a ddPCR method to detect SDC2 gene methylation and evaluate the diagnostic value of SDC2 gene methylation.

**Methods:**

First, a ddPCR method was developed to measure SDC2 methylation in stool samples collected from 51 cases of normal, 23 cases of adenoma, and 86 cases of CRC. Subsequently, a meta-analysis of existing studies was conducted to judge the diagnostic value of SDC2 gene methylation in CRC. PUBMED, EMBASE, Web of Science, and Scopus databases were searched for relative studies. Meta-analysis was performed using Meta Disc 1.4 and STATA 15.0 software.

**Results:**

The ddPCR showed that the linearity, sensitivity, and specificity for the detection of SDC2 gene methylation could be down to 0.1% methylation level and 5 ng of methylated DNA input. In 109 cases of CRC, 107 cases could be detected, and the sensitivity was 98.17%. The median value of the percentage of methylated reference (PMR) in colorectal adenoma and CRC patients was significantly higher compared with the normal individuals (*p* < 0.001). In addition, we found that the PMR value was associated with the clinical staging of CRC. The difference of PMR in stage II and stage IIIA was statistically significant (*p* < 0.05). Moreover, the meta-analysis showed that 11 out of 87 studies were identified to report the feasibility of SDC2 gene methylation as a method to diagnose early CRC. The pooled sensitivity and specificity of SDC2 gene methylation test for CRC were 0.80 [95% *CI* (0.68–0.88)] and 0.93 [95% *CI* (0.91–0.94)], respectively. The pooled diagnostic odds ratio (DOR) and area under curve (AUC) were 52.46 [95% *CI* (30.43–90.45)] and 0.94 [95% *CI* (0.92, 0.96)], respectively.

**Conclusions:**

The ddPCR method was more sensitive and convenient to detect SDC2 gene methylation, and the pooled analysis showed that methylated SDC2 was a valuable biomarker for the non-invasive detection of CRC.

## Introduction

Colorectal cancer (CRC) is the third most common cancer diagnosed worldwide. Every year, more than 945,000 people develop CRC globally, leading to about 492,000 CRC-related deaths (1). If CRC metastasizes, the average 5-year survival rate is estimated to be below 10%, while such rate may be as high as 90% if CRC can be detected in the early stage (2). The importance of diagnosis tools for early detection of CRC has been recognized worldwide. At present, colonoscopy is considered the “gold standard” for CRC diagnosis. It can show lesions more accurately and remove lesions at the same time, but it is invasive and associated with unpleasant intestinal preparation and the risk of intestinal perforation (3). The immunochemical fecal occult blood test (iFOBT), a non-invasive CRC diagnosis approach that uses antibodies against human globulin, has reduced CRC mortality by 15–33% (4, 5). However, this test is characterized by frequent false-negative and false-positive results (6). Therefore, it is urgently necessary to develop a non-invasive and more accurate diagnosis method to promote an early diagnosis of CRC.

DNA methylation is a major manifestation of epigenetics, involving a wide range of disease, such as Alzheimer's disease, diabetic nephropathy, and cancer (7). Abnormal methylation is the core of carcinogenesis, which usually leads to gene expression defects (8). Methylation of tumor suppressor genes is an early event in many tumors and may be one of the first changes related to tumorigenesis (9, 10). Many studies have identified specific DNA methylation sites, such as SEPT9, as biomarkers for CRC detection (11). Methylation status is tissue-specific and constant between several tissue types in different patients. Therefore, gene methylation has certain advantages as a biomarker for cancer detection (12). Brock's study found that methylation in the promoter regions of p16, CDH13, APC, and RASSF1A was associated with the early recurrence of lung cancer (13). Sobhani's study demonstrated that hypermethylation of the Wif1 promoter, the gene regulating the Wnt pathway, serves as diagnostic marker for early CRC (14). However, the value of methylation biomarkers in circulating tumor DNA (ctDNA) in CRC diagnosis and early detection needs further study. Syndecan-2 (SDC2), also called fibroglycan, encodes a transmembrane (type I) heparan sulfate proteoglycan. Hypermethylation of SDC2 has been reported in malignant glioma (15). SDC2 has been identified as a new potential epigenetic biomarker, which can be used to detect CRC using the CpG microarray method. Moreover, SDC2 shows a very high frequency of methylation even in the early stage. Therefore, SDC2 methylation can be used as a potential biomarker for the early detection of CRC.

To further confirm the diagnostic value of SDC2 gene methylation in CRC, we first developed a new technology of droplet digital PCR (ddPCR) to detect SDC2 gene methylation to improve the sensitivity of methylation detection. The principle of this method is that a DNA sample is divided into more than 10,000 droplets, and PCR amplification of template molecules takes place in each droplet (16). The simple readout of droplet partitions as a binary code of one (positive) and zero (negative) represents the “digital” aspect of the technique, and when the final droplet number is more than 10,000, the associated data fits a Poisson distribution (17). This method can be used to directly and simply calculate the copy number of DNA in the sample without a standard curve. Since ddPCR is an end-point PCR approach, it is not affected by the change of reaction efficiency. The high precision of this technology does not require repeated holes, which saves samples and time, and it also effectively allows the accurate quantitation of precious samples (18). In recent years, ddPCR has been more and more used in clinical practice because it can detect and quantify rare alleles more reliably, and it has the advantages of simplicity and rapidity (19). However, there are no reports about the use of ddPCR in the detection of SDC2 gene methylation and its application in the diagnosis of CRC. Therefore, we used ddPCR to detect SDC2 gene methylation and analyzed its application value in patients with CRC. Then, we conducted a meta-analysis of relevant studies to further study the diagnostic value of SDC2 gene methylation in CRC.

## Methods and Materials

### Patient and Sample Collection

All patients with CRC were from the Qilu Hospital of Shandong University. This study was approved by the Medical Ethics Committee of Qilu Hospital of Shandong University, and all subjects gave written informed consent to participate. Briefly, stool samples (~5 g) were collected from 43 patients with CRC, 23 patients with adenoma, and 51 normal individuals before bowel preparation. Another 43 CRC cases were received 1 week after colonoscopy but before surgery. The specimens were kept in 15 ml preservative buffer, followed by immediate storage at −80°C.

### DNA Extraction and Quantification

A QIAamp DNA Mini Kit (Qiagen) was used to extract DNA from stools according to the manufacturer's instructions. NanoDrop One (Thermo Scientific) was adopted to determine the concentration of DNA.

### Sequence-Specific Capture

Each capture reaction was carried out by adding 300 μl of stool DNA to an equal volume of 6 mol/L guanidine isothiocyanate solution (Sigma). The SDC2 gene capture probe was CGGTACTCTGCTCCGGATTCGTGTGC (20). After incubation at room temperature for 4 h, 50 μl prepared Dynabeads M-280 streptavidin (Thermo Fisher Scientific) was added to the solution, and the mixture was incubated at room temperature for 1 h. The bead/hybrid capture complexes were then washed two times with 1 × wash buffer (1.0 mol/L NaCl, 5 mmol/L Tris-HCl pH 7.5, and 0.5 mmol/L EDTA) and then eluted with 50 μl nuclease-free water containing 20 ng/ml transfer RNA (Sigma). Target gene SDC2 was captured in one reaction.

### Bisulfite Conversion of DNA

DNA was bisulfite-treated using EZ DNA Methylation-Gold^TM^ Kit (Zymo Research) according to the manufacturer's instructions. Briefly, DNA was added to the bisulfite treatment reaction. The sample tube was placed in a thermal cycler with the following steps: 98°C for 10 min; 64°C for 2.5 h, and storage at 4°C for up to 20 h. After the final wash step, DNA was eluted with 10 μL M-Elution buffer. The DNA transformed by sodium bisulfite should be used as soon as possible or stored at −80°C for later use. Repeated freezing and thawing were strongly discouraged as >3 freeze-thaw cycles could lead to DNA fragmentation that impaired PCR amplification.

### Droplet Digital PCR

Methylated copies of the SDC2 gene and the reference gene C-LESS (insensitive to CpG methylation status) were quantified using the QX200™ AutoDG Droplet Digital™ PCR System (Bio-Rad). Supplementary Table 1 shows the primer and probe sequences of SDC2 (21) and C-LESS (22) for this assay. The SDC2 reaction mixture consisted of 1x ddPCR Supermix for Probes (Bio-Rad), 900 nM of each primer, 250 nM of the probe, and 20 ng bisulfite-converted DNA template. The C-LESS reaction mixture consisted of 1x ddPCR Supermix for Probes (Bio-Rad), 900 nM of each primer, 500 nM of the probe, and 20 ng bisulfite-converted DNA template. Samples were loaded into the DG32^TM^ Automated Droplet Generator Cartridges (Bio-Rad). Then, the droplets were transferred to a 96-well PCR plate and placed into a C1000 TouchTM Thermal Cycle with 96-Deep well Reaction Module (Bio-Rad). Supplementary Table 2 lists the PCR cycling conditions. Data were analyzed using the QuantaSoft software (Bio-Rad). For each experiment, the following control samples were included: positive control well [Universal Methylated DNA (EMD Millipore)], negative control well [Universal Unmethylated DNA (EMD Millipore)], and non-template-control (NTC) well. The number of positive droplets in the negative control wells should be zero, which indicated the optimal specificity of the assay. In addition, NTC wells should also produce zero positive droplets, which reflected optimal laboratory practices. To obtain a measure of SDC2 gene methylation for each sample, the percentage of methylated reference (PMR) was the ratio of SDC2 to C-LESS in DNA extracted from fecal sample compared with the ratio of SDC2 to C-LESS in positive control (fully methylated DNA).

For quality control purposes, samples that generated <100 positive droplets per well for the C-LESS reaction were excluded.

### Real-Time Methylation-Specific PCR (QMSP)

Real-time methylation-specific PCR (qMSP) was used to detect SDC2 methylation in DNA samples from 43 stool specimens of CRC. PCR was conducted in a 25-μl reaction system consisting of 400 nmol/L of each primer, 200 nmol/L of each probe, 5 mmol/L Mg^2+^, 400 mmol/L dNTPs, 0.1 U/ml GoTaq Hot Start Polymerase (Promega), and 1 × buffer. For cell line and tissue samples, 1 μl bisulfite-converted DNA was added to the PCR reaction, while 5 μl bisulfite-converted captured stool DNA was used for stool samples. PCR was performed on a LightCycler 96 (Roche Diagnostics). Briefly, after an initial denaturation step at 95°C for 5 min, the amplifications were carried out with 10 cycles at a melting temperature of 95°C for 20 s, an annealing temperature of 62°C for 30 s, and an extension temperature of 70°C for 30 s, followed by 40 cycles at a melting temperature of 95°C for 20 s, an annealing temperature of 58°C for 60 s, and an extension temperature of 72°C for 30 s. Finally, a cooling step at 37°C for 30 s was conducted. Each plate consisted of bisulfite-treated DNA samples, positive and negative controls, and water blanks.

### Statistical Analysis

In the present study, GraphPad Prism 8 software was used to analyze the data. Pearson's correlation coefficient was used to verify the linearity of ddPCR detection. Non-parametric Kruskal–Wallis test was adopted to compare ctDNA concentration among different groups. The value of *p* < 0.05 was considered statistically significant.

### Meta-Analysis Methods

#### Study Selection Criteria

Inclusion criteria were set as follows: the techniques and target gene were clearly stated in articles; the target gene was verified by the detection of tumor samples; and sufficient data to construct a diagnostic table, such as true positive (TP), false positive (FP), false negative (FN), and true negative (TN).

Exclusion criteria were set as follows: experiments based on cell lines or animal models; studies were not written in English; and duplicate publications, reviews, letters, technical reports, case reports, or comments.

#### Search Strategy

The databases, such as EMBASE, PUBMED, Web of Science, and Scopus were searched using the keywords “SDC2,” or “syndecan-2,” and “colorectal cancer,” or “colorectal carcinoma,” or “colorectal tumor” to identify all relevant studies. Titles and abstracts of the articles identified through the keyword search were screened against the study selection criteria. Potentially relevant articles were retrieved for the evaluation of full texts.

#### Quality Assessment

The quality assessment of the articles was estimated using the revised Quality Assessment of Diagnostic Accuracy Studies 2 (QUADAS-2) guidelines. With signaling questions, the risk of bias and concerns regarding applicability were judged as “yes,” “unclear,” and “no.”

#### Data Extraction

Studies included throughout the process were evaluated by two reviewers, and in the case of disagreement, consensus could be reached through discussion between authors or submission to a third reviewer. The following information was extracted: (1) general information and relevant clinical information of the literature: such as title, country, year of publication, and author; and (2) diagnostic parameters of the literature: the detection value of SDC2 gene methylation and its extracted diagnostic four grid parameters, such as TP, FP, TN, and FN.

#### Meta-Analysis

First, the four grid table data, the author, and the year extracted from the studies were input into Stata15.0 software to estimate the pooled sensitivity, specificity, positive likelihood ratio, negative likelihood ratio, diagnostic odds ratio (DOR), and 95% *CI*. Statistical heterogeneity was assessed by *I*^2^ statistics. Generally speaking, *I*^2^ <25% indicated small heterogeneity, *I*^2^ between 25 and 70% reflected medium heterogeneity, and if *I*^2^ > 70%, it was considered to have high heterogeneity. Forest plots for the pooled sensitivity, specificity, positive likelihood ratio, negative likelihood ratio, and DOR of SDC2 gene methylation were generated for detecting CRC. Summary receiver operating characteristic (SROC) curves were plotted to assess the accuracy of SDC2 gene methylation for the detection of CRC. A *Z*-test was applied to examine the statistical difference of the areas under SROC curves (AUC).

In addition, the subgroup analyses were performed to estimate the effect of sample sources on the diagnostic performance of SDC2 gene methylation and explore heterogeneity using Meta Disc 1.4 (when the number of intergroup studies was <4, Stata 15.0 could not be used for the subgroup analysis). The presence of publication bias was tested by Deeks' funnel plot analysis.

All the analyses were conducted using Meta-Disc software 1.4 and Stata 15.0. Statistical tests presented were two-sided, and a *p* < 0.05 was considered statistically significant.

## Results

### Development of DdPCR Assay for the Detection of SDC2 Gene Methylation

**Figure 1:**
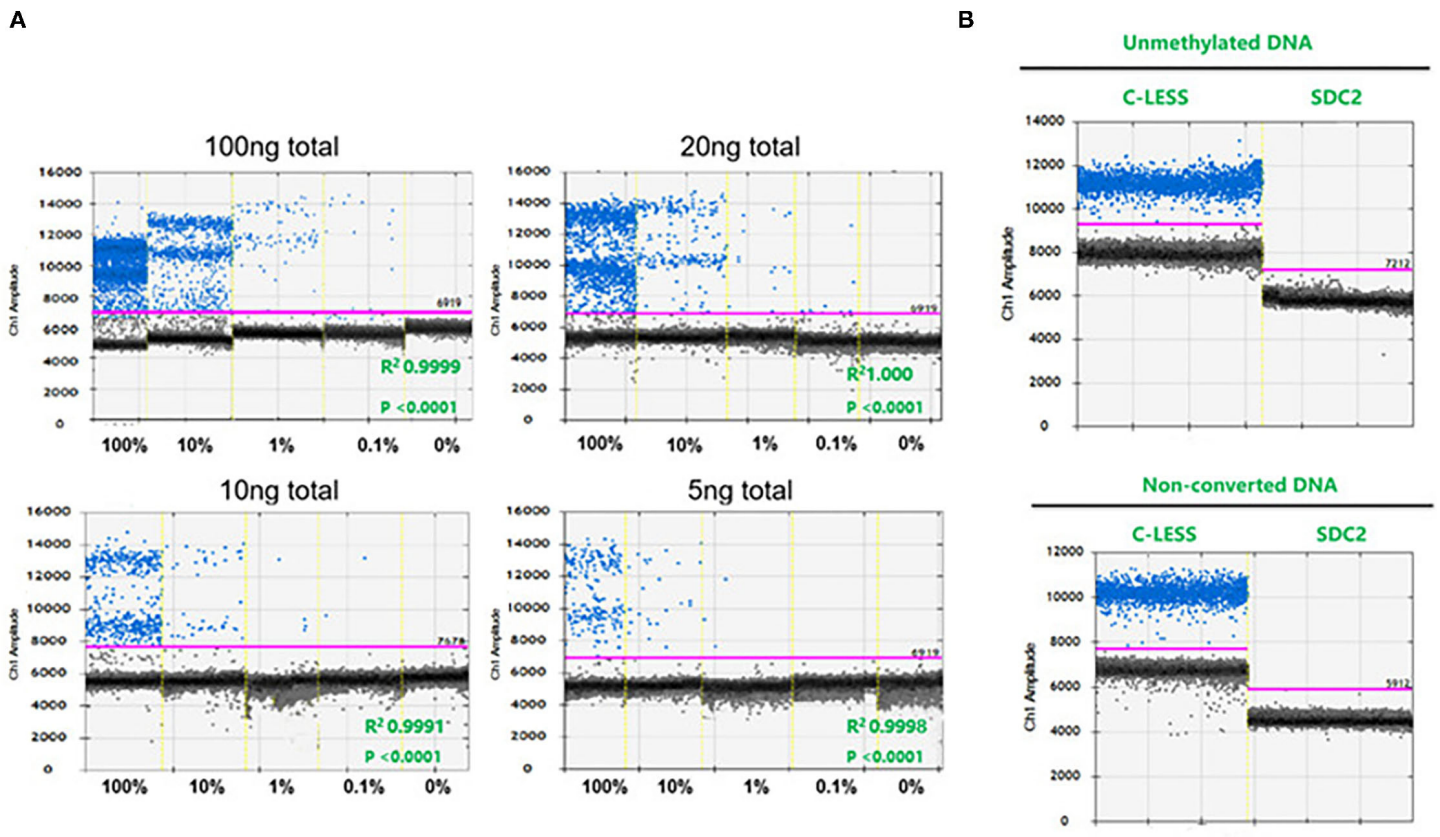
The sensitivity, specificity, and analytical range of the SDC2 gene methylation by droplet digital PCR (ddPCR) assay. **(A)** Serial 1:10 dilution of bisulfite-converted fully methylated control DNA (*n* = 3 independent replicates). **(B)** Unmethylated control DNA and non-converted DNA.

In the present study, we primarily aimed to evaluate the performance of ddPCR in detecting SDC2 methylation in the terms of sensitivity, specificity, and analytic range. The sensitivity and analytical range of SDC2 methylation analysis were determined by measuring the lower limit of detection. We used four different DNA quantities, such as 100, 20, 10, and 5 ng. Additionally, we prepared 10-fold serial dilutions of a fully-methylated control DNA in the back of fully unmethylated control DNA. The assay could be performed with an input DNA amount of as low as 5 ng, and the results showed good linearity over the titration series (100, 10, 1, 0.1, and 0%). Methylation of SDC2 could be detected down to 0.1% by ddPCR. The ddPCR could cope with a reduced amount of input DNA (Figure 1A). Supplementary Figure 1 shows the copies/well of various methylation levels over a range of concentrations. We chose 20 ng as the amount of DNA input. No positive droplets were detected when unmethylated control DNA or non-bisulfate-treated fully-methylated DNA was used as the input (Figure 1B), confirming the specificity of the assay for methylated DNA.

### High Sensitivity of DdPCR Assay for the Analysis of SDC2 Gene Methylation

**Figure 2:**
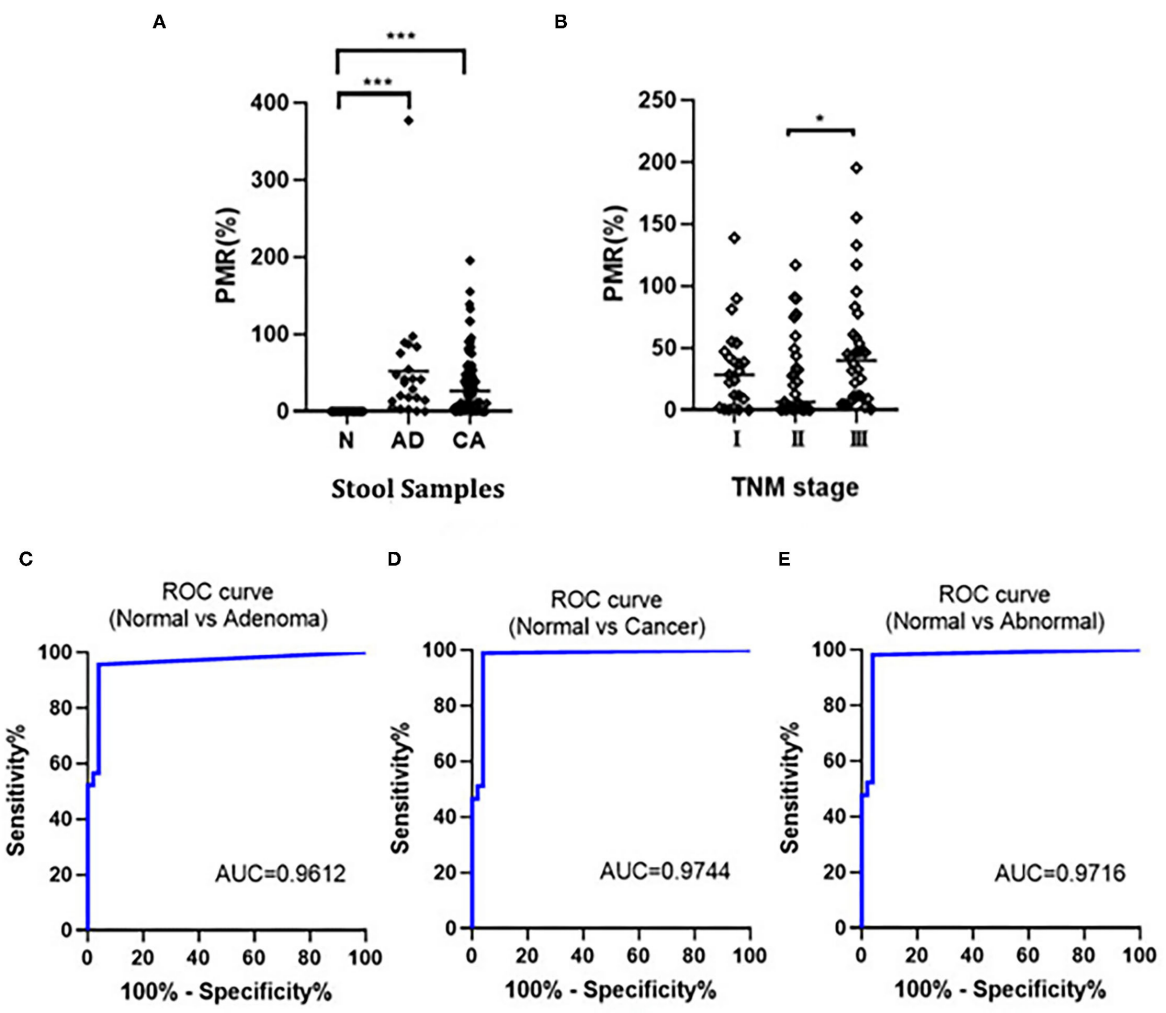
**(A)** Comparison of SDC2 percentage of methylated reference (PMR) in normal (N), colorectal adenoma (AD), and colorectal cancer (CRC) (CA). ***p < 0.001; **(B)** Correlation of SDC2 PMR in stool samples with CRC stages. *p < 0.05; **(C–E)** Receiver operating characteristic (ROC) curves to verify the diagnostic performance of SDC2 gene methylation.

We detected the methylation of the SDC2 gene in stool samples (51 cases of normal individuals, 23 cases of patients with adenoma, and 86 cases of patients with CRC) using ddPCR, and the methylation of SDC2 promoter was calculated as PMR using fully-methylated control DNA as a reference. The results showed that in 109 cases of CRC, 107 cases could be detected, and the sensitivity was 98.17%. The median value of PMR in colorectal adenoma and patients with CRC was significantly higher compared with the normal group (*p* < 0.001). There was no statistically significant difference between colorectal adenoma and patients with CRC (Figure 2A). Moreover, we found that the PMR value was associated with the clinical staging of CRC. The difference of PMR in stage II and stage III was statistically significant (Figure 2B). No significant relationships were observed between SDC2 methylation and clinical features, such as age, sex, tumor size, and tumor location (unpaired *t*-test, *p* > 0.05; Table 1). Then, we conducted ROC curves and calculated the AUC to verify the diagnostic performance of SDC2 gene methylation by ddPCR on CRC. The results showed that the AUC was 0.9612, 0.9744, and 0.9716 for discriminating normal from adenoma, cancer, and abnormal, respectively (Figures 2C–E). This finding suggested that SDC2 gene methylation possessed important diagnostic value for CRC. The result of qMSP detection showed that 29 of 43 patients with CRC were positive, and the sensitivity was 67.44%. Therefore, the method of ddPCR was better than qMSP.

**Table 1:** PMR results according to the clinical characteristics of colorectal cancer patients in this study.

**Characteristics (number)**	**PMR (%) Median (25th and 75th percentile)**	***P***
**Sex**		
Male (64)	20.27 (3.11, 48.95)	0.48
Female (45)	29.34 (9.84, 54.59)	
**Age**		
≤ 50 (29)	42.15 (7.430, 56.79)	0.35
>50 (80)	20.35 (2.850, 47.78)	
**Tumor size**		
≤ 4 (54)	26.80 (4.085, 48.74)	0.87
>4 (55)	28.72 (4.170, 59.78)	
**Location**		
Left (96)	29.03 (5.623, 54.90)	0.06
Right (13)	5.70 (0.460, 40.51)	
**TNM stage**		
I (23)	28.35 (1.920, 47.11)	0.03
II (27)	6.640 (0.820, 43.63)	
III (35)	39.97 (10.62, 60.28)	

### Literature Search Result and Quality Assessment for Meta-Analysis

**Figure 3:**
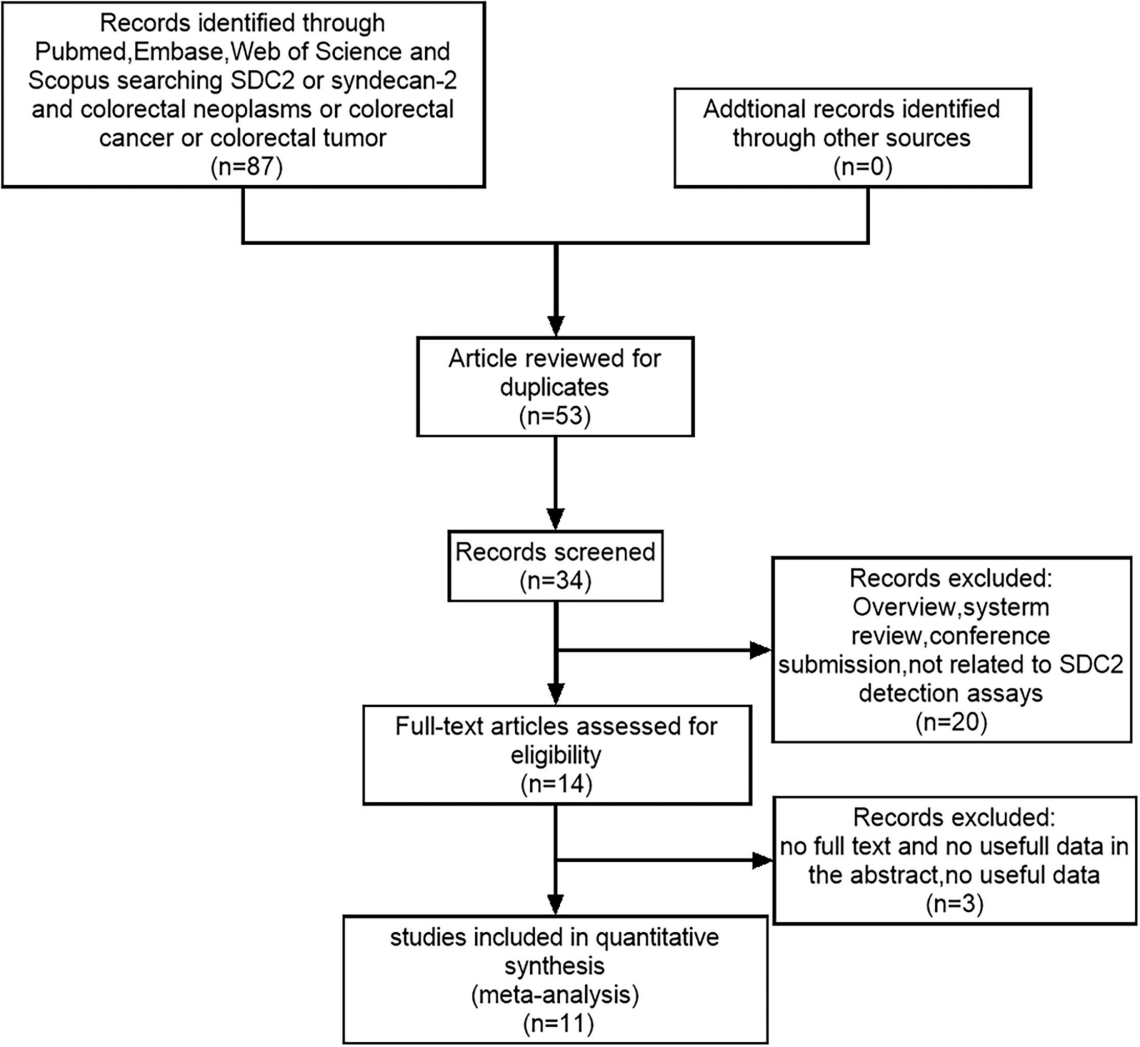
The PRISMA flow diagram for literature selection from relevant studies for this meta-analysis. The design of the diagram and the screening of the literature were based on the PRISMA statement for reporting meta-analysis.

Figure 3 shows that 91 studies were identified from the initial literature search. After the removal of 57 duplicates, the first round of title and abstract review was conducted, and 20 articles were excluded because they were overview, system review, and conference submission, or not related to SDC2 gene methylation detection for CRC. For the 14 remaining articles, a full-text review was conducted, and three of them were excluded due to the following reasons: no full text, no useful data in articles, and articles in Chinese. Finally, 11 eligible studies were included in the final review. The total number of cases included was 2,523, the minimum number of cases included was 64, and the maximum number was 490. Colonoscopy was used as the gold standard in all studies. Table 2 lists the characteristics and diagnostic parameters of the included studies. Moreover, we assessed the quality of the 11 articles according to the QUADAS-2 assessment tool (Supplementary Figure 2).

**Table 2:** Characteristics and diagnostic parameters of the included studies.

**Author**	**Country**	**Year**	**Sample sources**	**Number of cases**	**Gold standard**	**Method**	**TP**	**FP**	**FN**	**TN**	**Sensitivity**	**Specificity**	**AUC**
An, S. and T. J. Oh	USA	2019	Serum	168	Colonoscopy	RT-qPCR	107	7	10	44	0.915	0.863	0.937
Chen, Y., et al.	China	2019	Serum	225	Colonoscopy	RT-qPCR	79	5	32	109	0.712	0.956	0.881
Rasmussen, S. L., et al.	Denmark	2017	Serum	295	Colonoscopy	RT-qPCR	47	6	146	96	0.244	0.941	0.887
Oh, T., et al.	South Korea	2013	Serum	256	Colonoscopy	RT-qPCR	114	6	17	119	0.87	0.952	0.927
Zhao, G., et al.	China	2019	Plasma	283	Colonoscopy	RT-qPCR	81	7	36	159	0.692	0.958	0.886
Bartak, B. K., et al.	Hungary	2017	Plasma	84	Colonoscopy	RT-qPCR	42	1	5	36	0.89	0.97	0.93
Han, Y. D., et al.	South Korea	2019	Stool	490	Colonoscopy	RT-qPCR	221	24	24	221	0.902	0.902	0.902
Niu, F., et al.	China	2017	stool	373	Colonoscopy	RT-qPCR	159	12	35	167	0.811	0.933	0.92
Oh, T. J., et al.	South Korea	2017	stool	72	Colonoscopy	RT-qPCR	45	2	5	20	0.9	0.909	0.933
Sun, M., et al.	China	2019	Stool	213	Colonoscopy	RT-qPCR	72	9	33	99	0.686	0.917	0.8
Park, Y. S., et al.	South Korea	2018	Bowel lavage fluid (BLF)	64	Colonoscopy	RT-qPCR	8	6	2	48	0.8	0.89	0.844

*TP. Ture Positive; FP, False Positive; FN, False Negative; TN, True Negative*.

### Diagnostic Accuracy of Methylated SDC2

**Figure 4:**
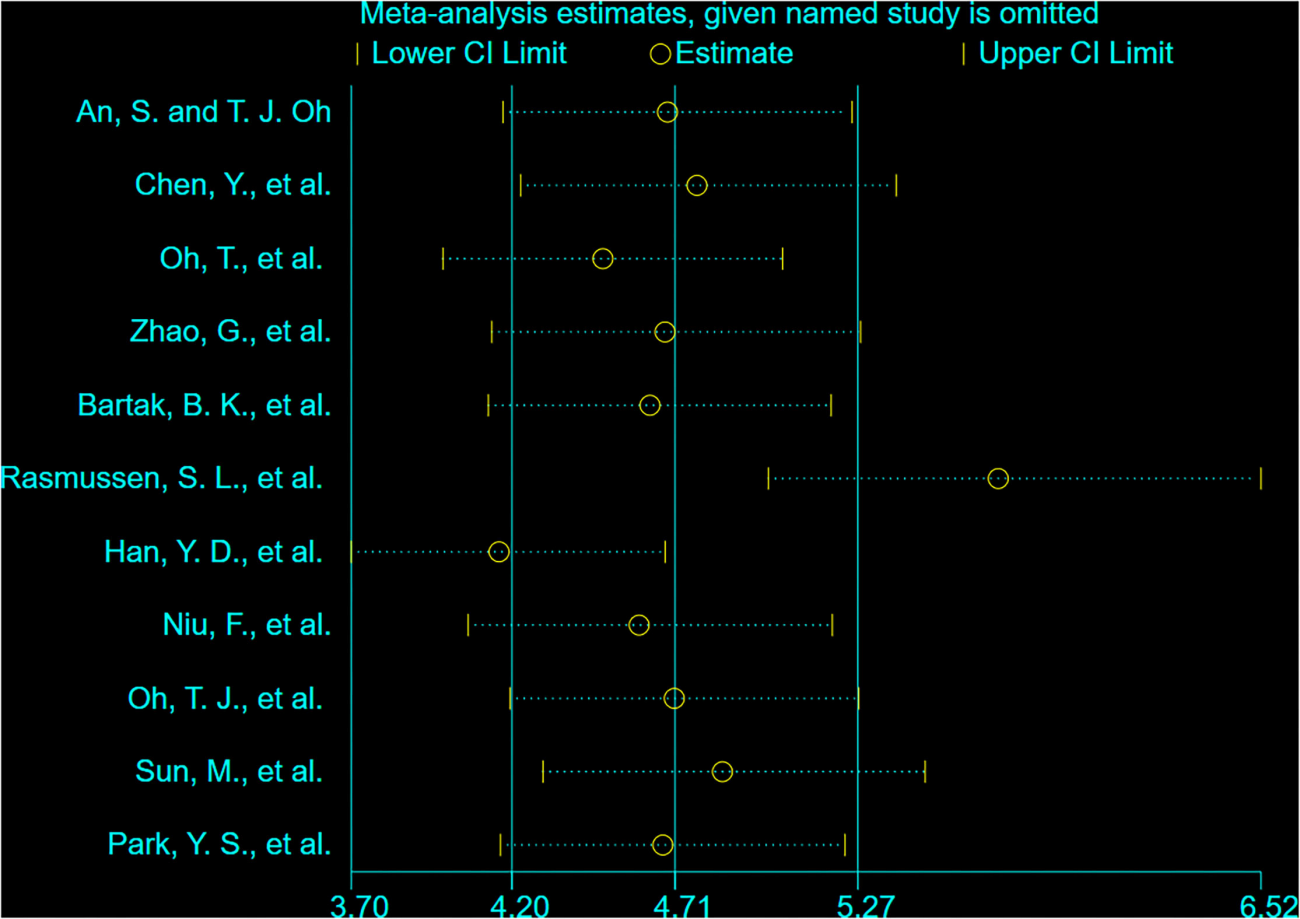
The sensitivity analysis of the included studies.

Supplementary Figure 3 summarizes the diagnostic performance of SDC2 for the detection of CRC. Meta-analysis was carried out on 11 included studies by using the random-effects model. The results showed that the combined sensitivity was 0.80 [95% *CI* (0.68, 0.88)], the specificity was 0.93 [95% *CI* (0.91, 0.94)], the positive likelihood ratio was 11.36 [95% *CI* (9.22, 14.01)], the negative likelihood ratio was 0.22 [95% *CI* (0.13, 0.35)], and the diagnostic ratio was 52.46 [95% *CI* (30.43, 90.45)]. The *I*^2^ of the combined sensitivity, combined specificity, positive likelihood ratio, negative likelihood ratio, and diagnostic ratio was 97.35, 54.25, 31.23, 98.57, and 100.00%, respectively, indicating that there was heterogeneity caused by non-threshold effect among the studies. Sensitivity analyses were performed to explore the sources of heterogeneity, and Figure 4 shows the results of sensitivity analysis. After the study of Rasmussen, S. L. was removed, the heterogeneity test was carried out again, and *I*^2^ was not decreased compared with the previous value.

### Subgroup Analysis

To evaluate the potential impact of factors, such as sample sources, and explore the sources of heterogeneity, we further analyzed the factors of sample sources in different groups (Supplementary Figure 4). Since there was only one study about SDC2 gene methylation test in bowel lavage fluid as CRC test, there was no subgroup analysis. The sensitivity of methylation detection of SDC2 gene in stool samples was 0.84 [95% *CI* (0.80–0.87)], in serum samples was 0.75 [95% *CI* (0.68–0.81)], and that in plasma samples was 0.63 [95% *CI* (0.59–0.67)] (*p* < 0.05). The negative likelihood ratio of methylation detection of SDC2 gene in fecal samples is 0.18 [95% *CI* (0.10–0.31)], in serum samples is 0.24 [95% *CI* (0.06–1.05)], and in plasma samples is 0.20 [95% *CI* (0.07–0.58)] (*p* < 0.05). The specificity, positive predictive value, and DOR obtained by subgroup analysis were not statistically significant (*p* > 0.05). Therefore, the sample source was the source of heterogeneity.

### Summary Operating Characteristics Analyses

**Figure 5:**
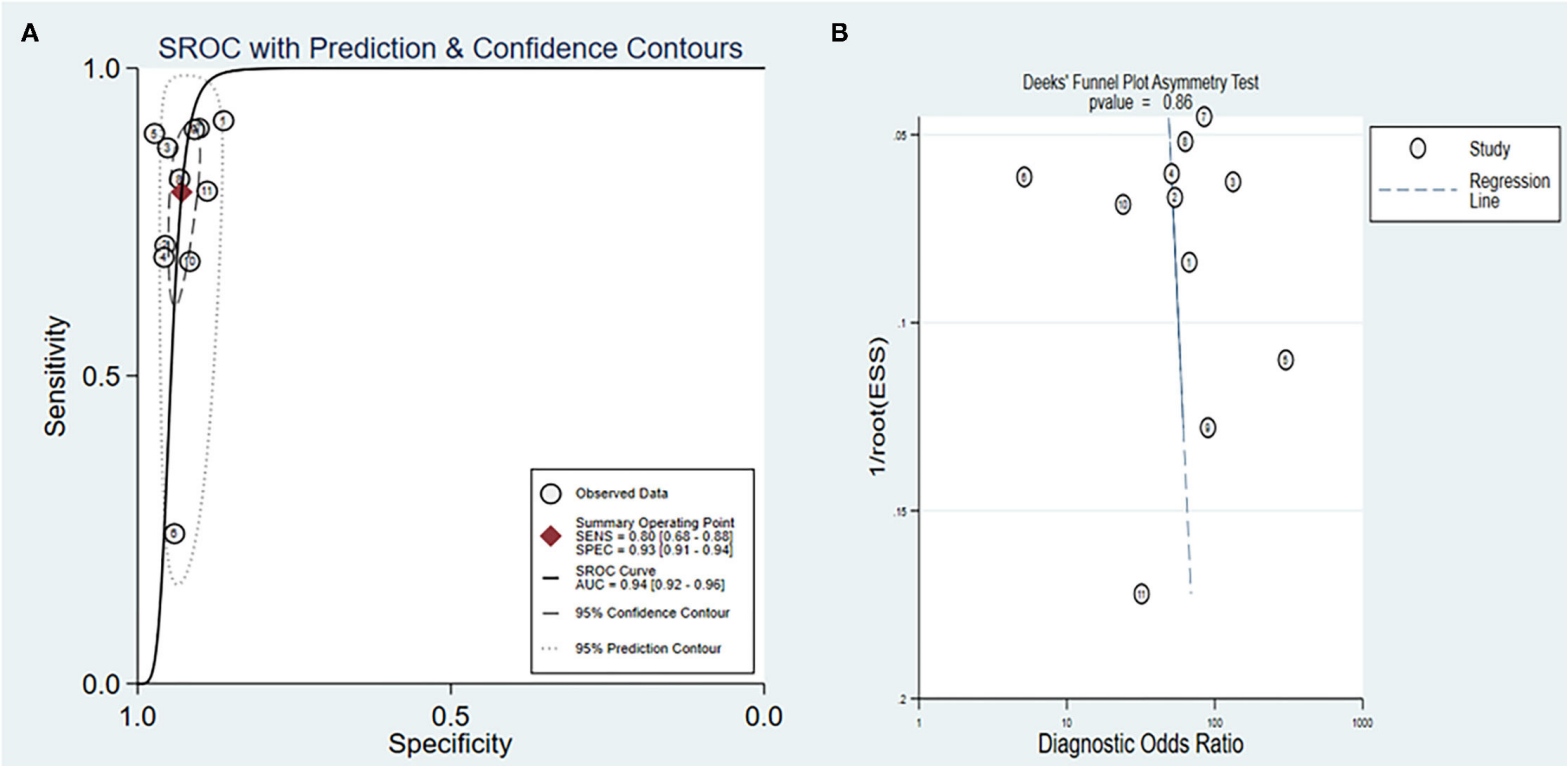
**(A)** Summary ROC (SROC) curve for SDC2; **(B)** Deeks' funnel plot asymmetry test for all studies included in this meta-analysis.

We further constructed SROC curves to verify the diagnostic performance of SDC2 gene methylation on CRC. The pooled AUC was 0.94 [95% *CI* (0.92, 0.96)] (Figure 5A), indicating that the methylation of the SDC2 gene had a significant diagnostic value as a detection indicator of CRC. The overall bias of the included studies was tested using the Deeks' funnel plot. The value of *p* of 0.86 indicated that the distribution of studies was symmetric, and there was no systematic bias across all studies analyzed in this study (Figure 5B).

## Discussion

Although surgical techniques, neoadjuvant chemotherapy regimens, and new therapeutic strategies of various drugs have developed greatly in the field of CRC treatment in recent years, many patients with CRC still develop into advanced or metastatic CRC, leading to the poor prognosis of these patients (23). Therefore, it is urgently necessary to find more effective biomarkers in the early diagnosis and targeted treatment of CRC.

PCR has been widely used to detect DNA methylation. The first-generation PCR is based on the PCR amplification of DNA digested by methylation-sensitive restriction enzyme (24). Then, there is a more flexible method, called MSP, which uses bisulfite to treat DNA fragments, converts unmethylated cytosine into uracil through deamination, and designs primers for the transformed DNA. The quantitative detection ability of DNA methylation has been greatly enhanced by fluorescence quantitative PCR (MethyLight). In MethyLight, primers and probes are designed for bisulfite transformation, and DNA sequence and quantitative information can be obtained in a real-time manner (25). Despite its advantages over MSP, MethyLight is still susceptible to PCR inhibitors and has limited sensitivity to detect rare methylation reactions. Therefore, the traditional qMSP is often unable to achieve the required precision and sensitivity for methylation detection (26). The next generation sequencing (NGS) can also detect rare mutations but with a lower technical sensitivity than ddPCR, unless (potentially costly) high sequencing depth is reached (27). Here, we described a novel and highly sensitive assay for the detection of methylated DNA based on ddPCR. We detected 109 tissues of CRC using ddPCR and calculated PMR using the FM control DNA as a reference. The results showed that in 109 cases of CRC, 107 cases could be detected, and the sensitivity was 98.17%. Additionally, we calculated PMR using the number of copies/20 μl well. The median value of PMR in colorectal adenoma and patients with CRC was significantly higher compared with the normal individuals (*p* < 0.001). Moreover, we found that the PMR value was associated with the clinical staging of CRC. The difference of PMR in stage II and stage III was statistically significant (*p* < 0.05). The above-mentioned results indicated that SDC2 gene methylation had a certain value in the diagnosis of patients with CRC. To further prove this point, we conducted a meta-analysis.

In this meta-analysis, 11 articles meeting the inclusion criteria were included through database retrieval and manual retrieval, with a total of 2,523 samples. According to the QUADAS-2 quality evaluation standard, the quality of all the literature included in the evaluation was of medium and high quality, and the bias mainly focused on the selection of research objects and the two parts of the diagnostic test to be evaluated. The combined sensitivity, specificity, and DOR of 11 studies were 80, 93, and 52.46%, respectively, and the positive likelihood ratio and negative likelihood ratio were 11.36 and 0.22, respectively. We found that the sensitivity of ddPCR (98.17%) to detect SDC2 gene methylation was much higher compared with the quantitative PCR (qPCR). The positive likelihood ratio was more than 10, and the negative likelihood ratio was <0.1, indicating that the index had high accuracy. In addition, the AUC of the combined ROC curve of SDC2 gene methylation for cancer diagnosis was 0.94. It is generally considered that AUC of 0.5–0.7 indicates low diagnosis accuracy, 0.7–0.9 indicates medium diagnosis accuracy, and above 0.9 indicates high diagnosis accuracy (28). It indicated that SDC2 gene methylation had high efficiency for cancer diagnosis. To evaluate the potential impact of factors including sample sources, we further analyzed the factors of sample sources in different groups. We found that the sample source was the source of heterogeneity. It also suggested that the accuracy of methylation of the SDC2 gene in fecal samples was higher compared with plasma, serum, and intestinal lavage samples.

When the subgroup analysis was conducted to compare the differences between sample sources, we found an interesting question. It is well known that the difference between serum and plasma is the lack of clotting factors and fibrinogen due to the solidification process. However, according to the results of subgroup analysis, the sensitivity of serum and plasma was 63 and 75%, respectively. At present, the consensus is that plasma samples are more suitable for ctDNA analysis compared with serum in clinical application (29). Although serum usually produces higher levels of cell-free DNA (cfDNA) (30), it may contain the higher concentrations of DNA released during the dissolution of circulating leukocytes (such as, neutrophils), which may reduce the relative proportion of ctDNA (25). Serum collection requires coagulation at room temperature, which also increases the risk of cytolysis and ctDNA degradation. If the detection of SDC2 gene methylation can be applied in clinical practice, plasma collected from patients may be preferable to serum samples. However, we could not make a definitive conclusion due to the limited number of samples. Subsequent studies on relevant aspects should be carried out for verification.

There are some limitations in this study. First, we only used CRC stool samples to detect SDC2 gene methylation. This accurate and sensitive method should be focused on the methylation detection of cfDNA (serum, plasma, or urine) in future studies, providing a greater value for clinical application. Second, the number of studies used for the meta-analysis is small. Besides, we have strict requirements on the data included in the literatures, some authors of related articles did not provide the original data. The limited number of samples in this study might affect the results of meta-analysis. Third, the purpose of this study was to explore the methylation of the SDC2 gene as a biomarker of CRC detection. However, the cancer pathological stages and the limited number of samples in this study might affect meta-analysis results. Finally, this study we only used single marker (SDC2) for CRC detection. Studying a multiplex of markers could give more strength to this study. For example, Mazouji, O's group and other researchers have demonstrated the diagnostic role of several non-invasive methylation biomarkers in CRC detection, such as WIF, NPY, PENK, SEPT9, VIM, Alx4, and others (31). The role of multi-target stool DNA testing in CRC diagnosis has attracted more and more attention. Imperiale, T. F's research showed that multi-target stool DNA testing can significantly detect more cancers in asymptomatic persons with an average risk of colorectal cancer (32).

## Conclusions

Collectively, SDC2 gene methylation had high efficiency in the diagnosis of CRC, which might be used as an important reference index for CRC detection. The results of subgroup analysis showed that the diagnostic efficiency of SDC2 gene methylation in feces was higher compared with other sample sources. However, more investigations are still required in future research. We found that ddPCR was more sensitive and convenient to detect SDC2 gene methylation.

## Data Availability Statement

The raw data supporting the conclusions of this article will be made available by the authors, without undue reservation.

## Author Contributions

CY and YaZ created the figure and wrote the manuscript. YW, ZZ, MZ, HW, WC, ZS, YX, XZ, and YiZ provided comments and suggestions. All authors have read and approved the final manuscript.

## Funding

This study was sponsored by the National Natural Science Foundation of China (81972005 and 81702815), the Natural Science Foundation of Shandong Province (ZR201910220159), Shandong Medical and Health Technology Development Project (2018WSB20002), and the Key Technology Research and Development Program of Shandong Province (2019GSF108247).

## Conflict of Interest

The authors declare that the research was conducted in the absence of any commercial or financial relationships that could be construed as a potential conflict of interest.

## Publisher's Note

All claims expressed in this article are solely those of the authors and do not necessarily represent those of their affiliated organizations, or those of the publisher, the editors and the reviewers. Any product that may be evaluated in this article, or claim that may be made by its manufacturer, is not guaranteed or endorsed by the publisher.

## Supplementary Material

Supplementary Figure 1 The copies/well of various methylation levels (100, 10, 1, 0.1, and 0%) over a range of concentrations (from 100 to 5 ng).

Supplementary Figure 2 The study quality of included studies.

Supplementary Figure 3 Forest plots for the SDC2 gene methylation tests on the detection of colorectal cancer (CRC). **(A)** Combined sensitivity and specificity forest plots. **(B)** Combined positive likelihood ratio and negative likelihood ratio forest plots. **(C)** Combined diagnostic ratio forest plots.

Supplementary Figure 4 Subgroup analysis of the detection of CRC by methylation of SDC2 gene from different sample sources, such as stool, serum, and plasma. **(A–E)** The pooled sensitivity, specificity, positive likelihood, and negative likelihood of SDC2 gene methylation from different sample sources, respectively.

Supplementary Table 1 The primer and probe sequences for assay.

Supplementary Table 2 ddPCR cycling conditions.
