# Supplementary figures and images for: The Application Value of Syndecan-2 Gene Methylation for Colorectal Cancer Diagnosis: A Clinical Study and Meta-Analyses
Source: Front Med (Lausanne). 2022 Mar 15;9:753545. doi: 10.3389/fmed.2022.753545 (PMC8964598; doi:10.3389/fmed.2022.753545)

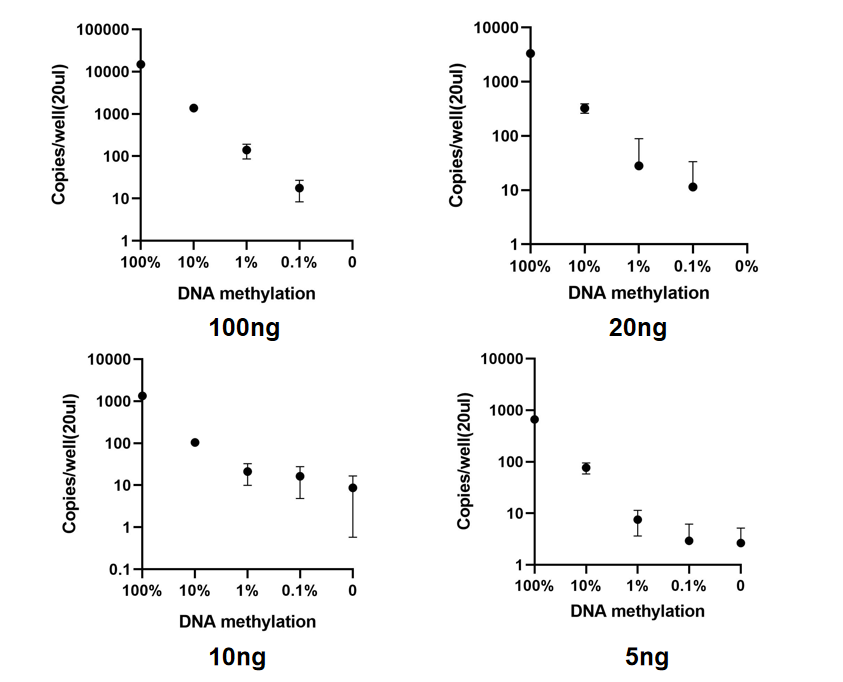

Supplement: Supplementary Figure 1 — The copies/well of various methylation levels (100, 10, 1, 0.1, and 0%) over a range of concentrations (from 100 to 5 ng). [file Data_Sheet_1.ZIP › Supplement Data Figure 1.tif]

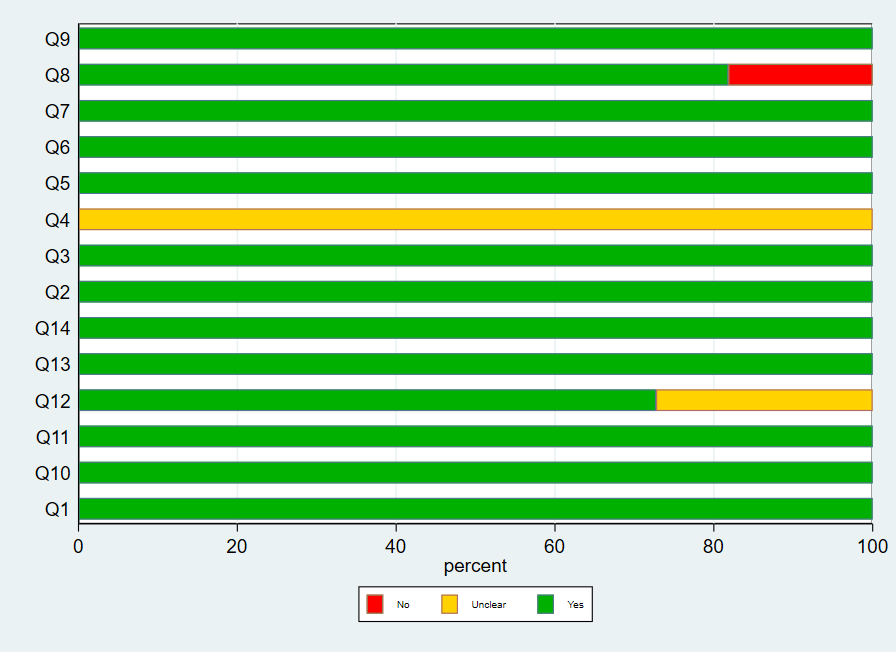

Supplement: Supplementary Figure 1 — The copies/well of various methylation levels (100, 10, 1, 0.1, and 0%) over a range of concentrations (from 100 to 5 ng). [file Data_Sheet_1.ZIP › Supplement Data Figure 2.tif]

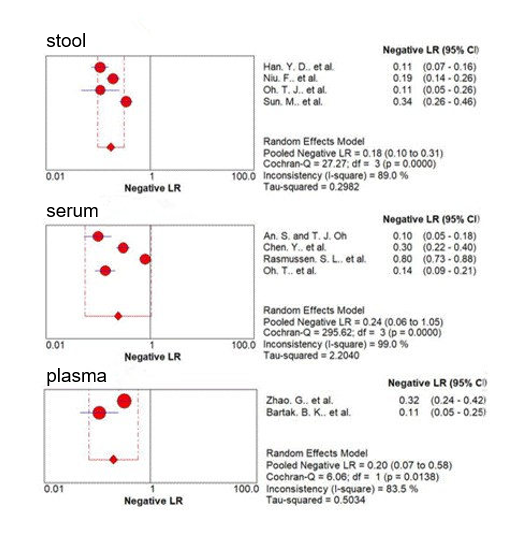

Supplement: Supplementary Figure 1 — The copies/well of various methylation levels (100, 10, 1, 0.1, and 0%) over a range of concentrations (from 100 to 5 ng). [file Data_Sheet_1.ZIP › Supplement Data Figure4D.tif]

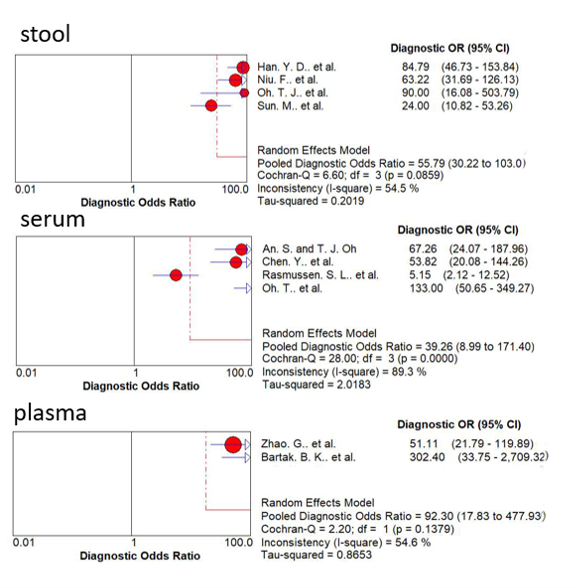

Supplement: Supplementary Figure 1 — The copies/well of various methylation levels (100, 10, 1, 0.1, and 0%) over a range of concentrations (from 100 to 5 ng). [file Data_Sheet_1.ZIP › Supplement Data Figure4E.tif]
